# Preserving identities in post-industrial Rust Belt cities: reconsidering Buffalo’s material memory

**DOI:** 10.1186/s40410-022-00158-1

**Published:** 2022-05-12

**Authors:** Miguel Guitart

**Affiliations:** Department of Architecture, University at Buffalo SUNY, Buffalo, NY 14214 USA

**Keywords:** Material identity, Material memory, Industrial preservation, Post-industrial architecture, Rust Belt cities, Buffalo infrastructure, Urban ecosystem

## Abstract

In the context of post-industrial Rust Belt cities, much needed investment seldom makes room for proper debate on the material memory and place identity embodied in the rich industrial legacy. However, a reflection on the intrinsic value of the vacant industrial structures of these cities leads to the unveiling of their most authentic identity. This identity is directly related to the material experience of their structures as it speaks to the qualities of a lasting presence in the collective memory. Post-industrial structures display material conditions that define their place character, constituting a negotiation between ruin and construction. This text contends that post-industrial materiality embodies necessary and strategic connections between past heritage and future interventions, implying the possibility of cyclical renovation in a context of reinforced identity. The text focuses on the potential of the existing post-industrial legacy of the city of Buffalo, NY, arguing that the material dimension that once defined its productive infrastructure frames the potential consolidation of its physical memory and future identity, and as such, a consolidation of its future growth.

## Introduction: from decay to resurgence

“In such spectacular urban scenes as the view down the Buffalo River toward the Ohio Street Bridge […] one can see that the combination of assured durability and long-sustained functional relevance has given concrete elevators a monumental longevity.” Reyner Banham.^1^

Vacant industrial structures generously dot the urban landscapes of Buffalo, NY. Other Rust Belt cities offer similar landscapes. Pittsburgh, Detroit, and Cleveland offer a high percentage of derelict structures that remain vacant and face the potential fate of demolition. As local Rust Belt economies improve, debates around the need for new buildings versus the preservation of existing ones emerge as part of a more sustainable global agenda. A greater reflection on the character and value of such structures may be conducive to effective adaptation, sustaining the historic identity of the cities that host them. The potential reutilization and reintegration of these structures can invigorate the social, cultural, and economic evolution of Rust Belt cities, consolidating the positive impact that such reintegration could pose to the architectural identity—and economy, as we will see later—of these cities. The present text contends that, despite the vicissitudes that emerge from other historic and economic considerations, post-industrial material memory, that is, the capacity that these material assemblies have to embody, record, and preserve a collective identity by means of a set of material qualities, determines the character and ultimately the place identity of these Rust Belt sites. This work focuses on the architectural identity of Western New York’s largest city, Buffalo. Through reintegration and reuse, the material legacy of Buffalo’s industrial infrastructures preserves the character of the city, and ultimately, of its own identity.

The evolution of industry and transportation, and the conflicts that arose between emerging and declining regional economies, induced a state of progressive abandonment of the once burgeoning cities of the Rust Belt. Years of divestment, exodus, stagnation, and neglect consumed America’s Large Lakes cities, resulting in a dramatic decrease of their overall prosperity.^2^ The built legacy of these industrial hubs soon loomed ghostly amidst abandoned lots and vacant shells. Disregarded by some as “tons of deserted, decaying concrete,”^3^ abjectly decaying fabrics faced uncertain futures. Richard Florida, an American urban theorist who focuses on social and economic theory, offers the following description: “For decades, the Rust Belt was synonymous with deindustrialization and economic decline. […] Images of shuttered factories and abandoned neighborhoods […] As factories moved to the suburbs, the Sunbelt, or off-shore, jobs and people followed. Those who could, moved away. Neighborhoods and entire cities lost their economic function and hollowed out.”^4^

In recent years, this context has started to show certain signs of change, giving way to a new, sometimes timid, resurgence, which has at times been termed as a ‘renaissance.’ After the financial crisis of 2008 and Detroit’s bankruptcy on July 18, 2013, the Large Lakes region seems to be slowly responding to new initiatives and contexts that have the potential to reshape some of its cities, physically, socially, and financially.^5^ While not without difficulties, a number of reasons may explain this slow, but steady awakening.^6^ Richard Florida coined the term “return migration” to relate to family relocation, which increased during the financial crisis of 2008, as affected individuals sought lower real estate costs. This circumstance has been exacerbated by the recent Covid-19 pandemic, which has fueled this type of migration to post-industrial cities as a response to national housing shortages, the desire for lower density living, and the expansion of remote working opportunities.^7^ As a result, initiatives of small-scale business growth in the fields of crafts, manufacturing, service, and agricultural-related fields have increased in number.^8^ As families and professionals have relocated and reinvented themselves after being directly affected by different national crises occurring between 2007 and 2020, a resurgence of local entrepreneurial opportunities has prompted a significant acceleration in ‘return migration.’ This tendency is illustrated by the population increase in cities such as Buffalo, which grew in 2020 for the first time in seven decades, as indicated by the 2020 census.^9^

**Fig. 1:**
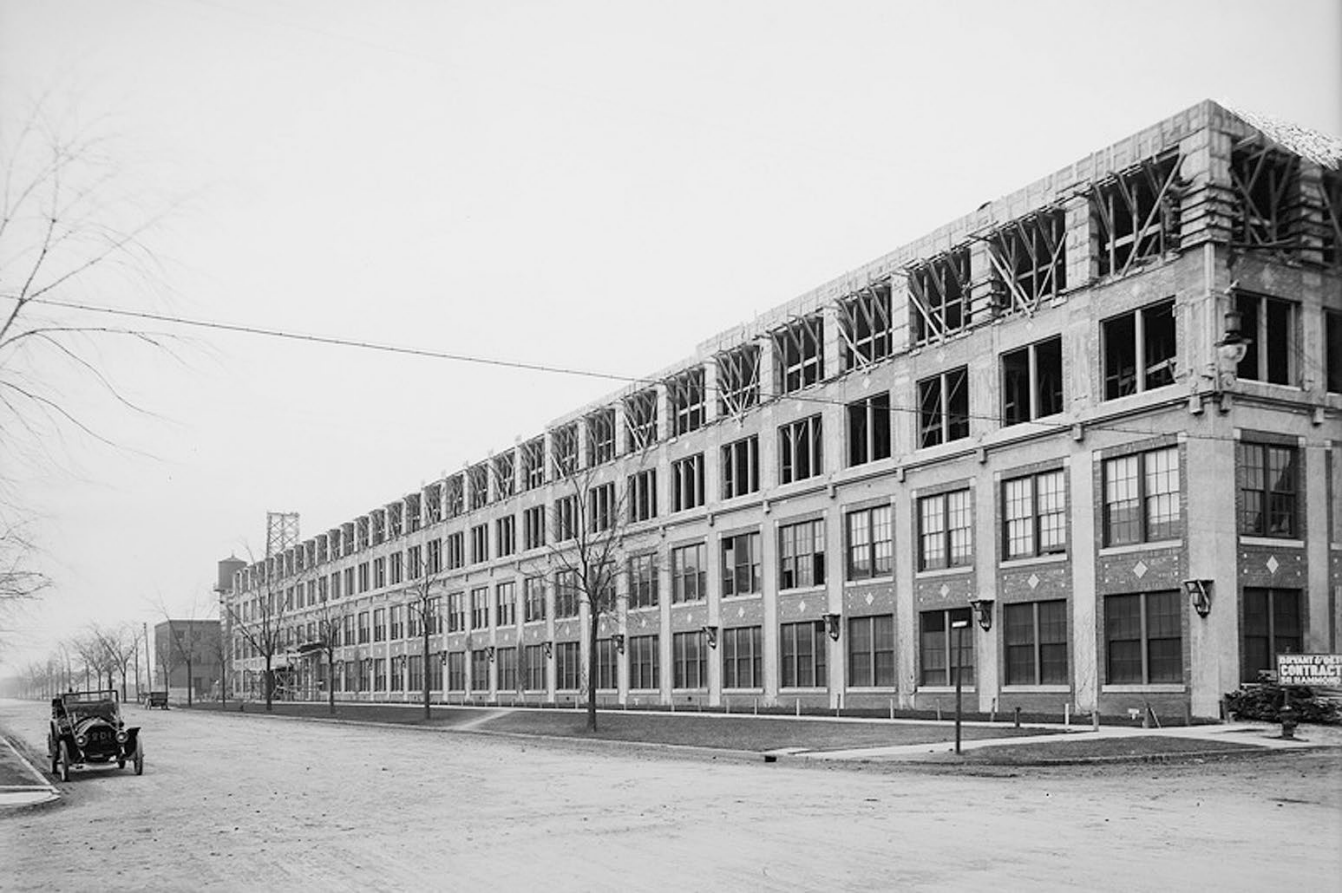
Packard Automobile Plant, Detroit, Michigan. Architect: Albert Kahn (Photo by unknown author)

Years of decay in Rust Belt post-industrial cities have, in fact, given way to renewed opportunities in infrastructural considerations. Cities that once represented unbridled leaders in American industrialization, such as Buffalo, Cleveland, Pittsburgh, and Detroit, have seen the recent squalor of their social and economic situations benefit from minorities, professionals, and entrepreneurs who, seeking new lives and professional paths, opt for alternatives to the traditional migration to larger hubs.^10^ These sites can be counted amongst the post-industrial hubs that have experienced some degree of renewed florescence through economic, architectural, and infrastructural reconfiguration. Small, local, and community-oriented initiatives in these cities weave into a renovated social, economic, and architectural fabric.^11^ Following the 2007 crisis, a steady pace of activity is infusing the way in which Rust Belt cities are revitalized and reconfigured.^12^ Vacant lots and crumbling abandoned structures become opportunities for growth, creativity, and alternative progress, offering a new context for urban, architectural, and social venues.^13^ (Fig. 1).

However, the influx of investment, social and architectural initiatives, and large-scale urban infrastructure projects in Rust Belt cities can occur with a disregard for local, historical and social assets. Developers and investors typically set aside the architectural implications of new interventions in the existing industrial inheritance, demonstrating little concern for the intrinsic historical, architectural, and material value that these structures hold for the city’s identity. The prevailing absence of intellectual and cultural ambition, qualified planning, and heritage discussion-making in real estate operations, leads to architectural decisions lacking contextual consideration. The demolition of significant structures typically gives way to unfortunate constructions dictated by short-term goals, clumsy in design and hasty in execution. The long-term consequences of erasing the character and identity of these places involves the general devaluation of the neighborhoods affected by these transformations. While not all new developments are careless and certainly not all existing industrial structures are worthy of being preserved, the urgency of investment over a carefully planned symbiosis with the existing city seems to justify speculative operations that disregard forms of collective memory. Mundane new constructions embody a lack of critical reflection regarding the specific material legacy of these sites. These architectural initiatives become missed design opportunities and irreparable losses that do not reckon the intrinsic material identity and place character of these cities.^14^

## Industrial structures as modern paradigms: a formal identity

**Fig. 2:**
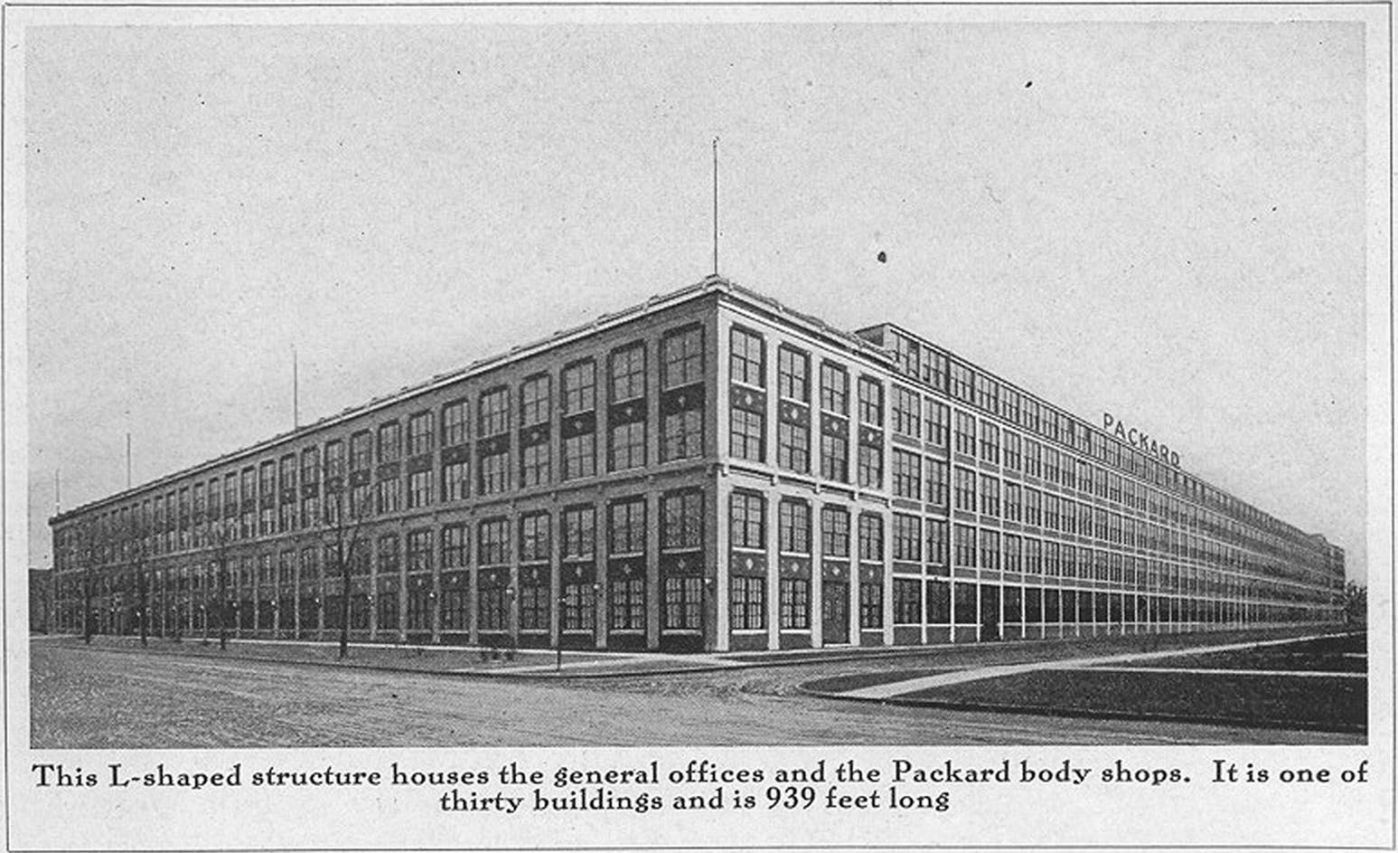
Packard Automobile Plant, Detroit, Michigan. Architect: Albert Kahn (Photo by unknown author)

The identity of a place is embedded in the perception that citizens have of their material context. The structures that define our places are characterized by a specific material presence, one that inhabitants relate to through their senses, and which distinguishes the identity of those places.^15^ These structures function as components of a larger continuum, in which the experience of the urban fabric is akin to a continuous combination of the physical qualities of an area as a whole, blurring the limits between construction and non-construction strategies. The material identity of a place refers to the agents that construct the physical imprint of a collective memory.^16^ (Fig. 2).

In Rust Belt cities, some of the most significant derelict infrastructures of today are the once thriving industrial constructions. As a whole, these constructions continue to configure the strong visual and material identity of their urban tapestries. This is exemplified by Buffalo’s grain silos and daylight factories, also termed ‘gridded spaces.’^17^ The grain silos on the Buffalo river remain a unique case of an age-old industrial infrastructure that acts as a defining element for citizens, visitors, and investors, conforming to the collective image of the city beyond its limits. As suggested by Dr. Lynda Schneekloth, the silos may be thought of as “negative icons to a glorified past,”^18^ but they constitute an integral part of the layers of memory that identify the city. Local curator Claire Schneider writes that the silos “loom with increasing disproportion over the surrounding environment. Possessing the dignity of age, while apparently slightly out of place, they stand as witnesses of the past.”^19^ These gigantic industrial structures emerge as the most remarkable presence of all post-industrial buildings in Buffalo, bringing to mind the distant memory of the ruins of ancient walls and temples of medieval cities, like “shards of their original splendour.”^20^

The impact that the grain silos have on Buffalo’s identity can be traced back to the Industrial Revolution at the end of the nineteenth century in Europe.^21^ These simple and functional forms were celebrated at the time among European architects, who published them widely in polemical comparison to a range of historic and contemporary predecessors, such as the Egyptian pyramids, Greek temples, automobiles, airplanes, and steamships. Dr. Keith Eggener has written extensively about identity in American architecture, explaining, for instance, how American modernism “took inspiration from local vernacular and industrial structures—from the clean lines, material economy, straight-forward construction, efficiency, and simple beauty of American factories. […] [In] eschewing the industrialist embrace of concrete and steel, […] architects were creating an architecture more genuinely functional.”^22^ In embracing this character, American modernism maintained a stronger local identity through the deployment of successful design solutions. Modern architects praised the silos as examples of modern functional design uncluttered by ornament, picturesque composition, or historical references. Hadas Steiner writes, “the fact that the forms of these structures had been generated by subjecting the behavior of grain to the needs of the transshipment rather than by recourse to the bygone conventions of architectural composition purified them from the contamination of style. Accordingly, structures of pedestrian origin were elevated from their status as mechanical contraptions to that of a generator of an aesthetic discourse—in other words, the low became high architecture.”^23^

One of the architects and theorists to first use images of American grain elevators was Walter Gropius. At the turn of the twentieth century, Gropius was working in the office of Peter Behrens, who would be the first architect to design a canonical industrial space, the AEG turbine factory in Berlin, built between 1908 and 1909. Gropius worked at Behrens’ office until 1910, when he left to work with his colleague Adolf Meyer. Together, Gropius and Meyer designed the iconic Fagus factory, in Alfeld on the Leine, Lower Saxony, in 1913, and the Deutscher Werkbund factory in Cologne, in 1914. Both buildings were largely influenced by the daylight factory typology, which, like the grain elevators, originated as a form of American industrial architecture and were widely published in European journals. Their open plans and extensive glazing inspired modernist architects. Not surprisingly, Gropius’ essay on modern architecture in the *Jahrbuch des Deutschen Werkbundes*, written in 1913, included photographs of Buffalo's grain elevators in addition to images of five American daylight factories.^24^ Gropius illustrated his remarks with photographs of the Washburn–Crosby complex in Minneapolis—today the Mill City Museum—and the Dakota elevator in Buffalo—built in the 1870s and demolished in 1965.^25^

**Fig. 3:**
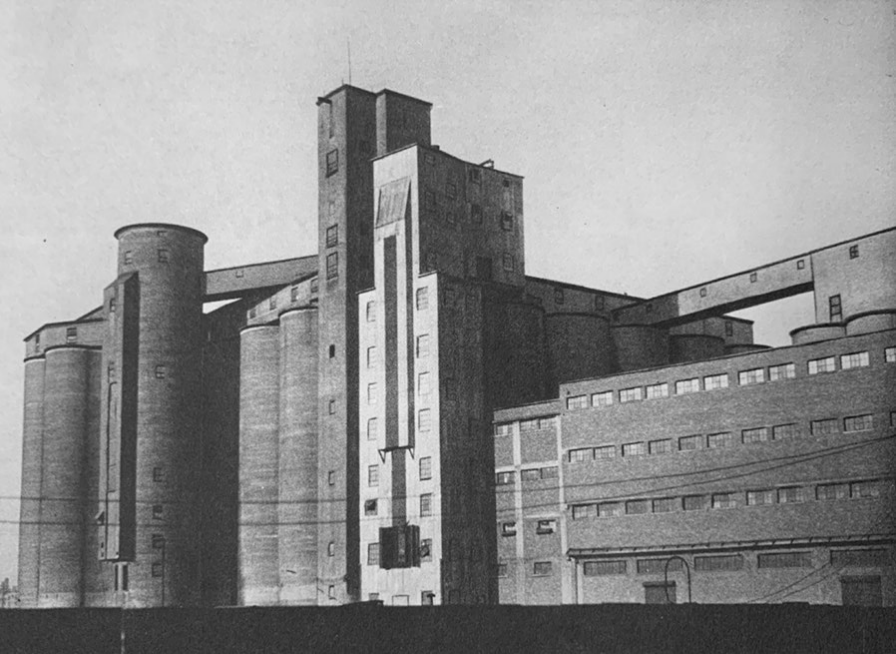
Grain silos. Buffalo, New York. Erich Mendelsohn. 1926. From his book *Amerika. Bilderbuch eines Architekten* (Berlin: Rudolf Mosse/Buchverlag, 1926)

**Fig. 4:**
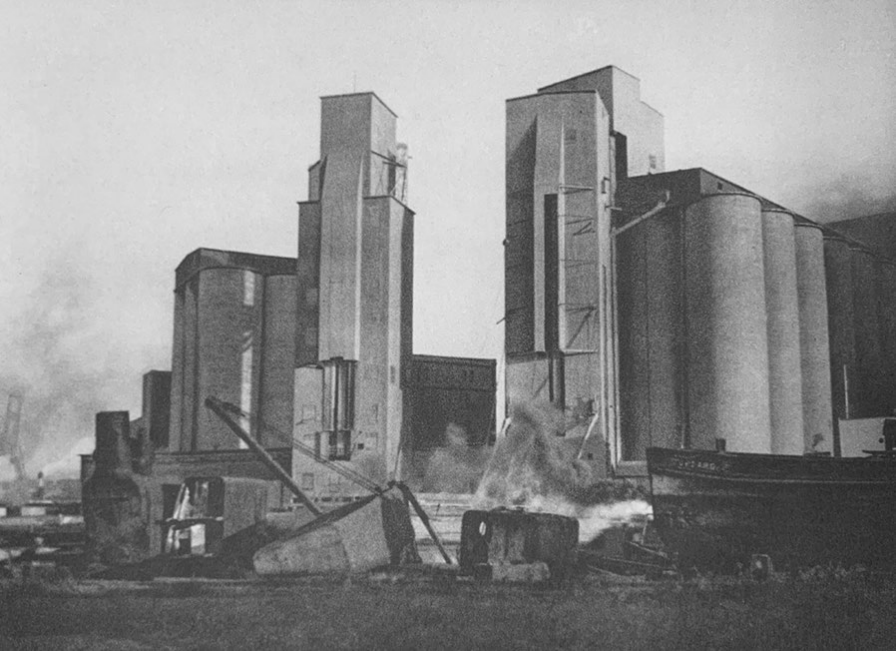
Grain silos. Buffalo, New York. Erich Mendelsohn. 1926. From his book *Amerika. Bilderbuch eines Architekten* (Berlin: Rudolf Mosse/Buchverlag, 1926)

**Fig. 5:**
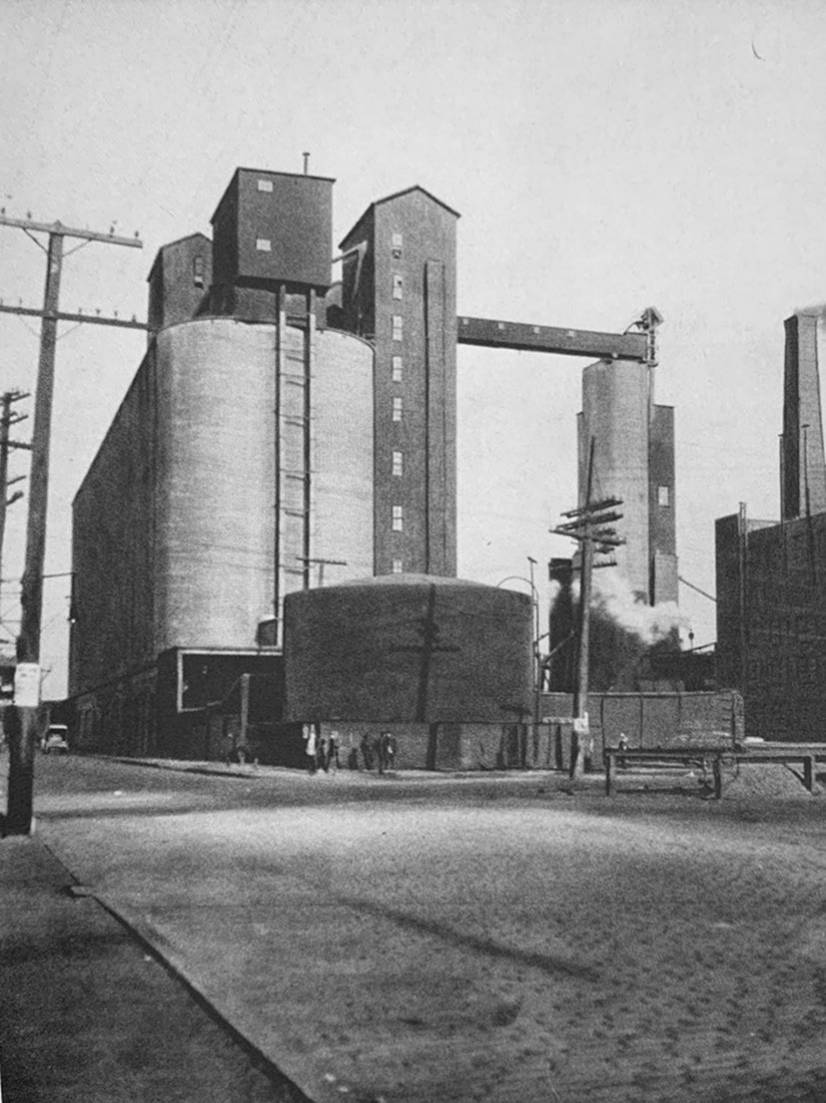
Grain silos. Buffalo, New York. Erich Mendelsohn. 1926. From his book *Amerika. Bilderbuch eines Architekten* (Berlin: Rudolf Mosse/Buchverlag, 1926)

A few years later, between 1921 and 1923, Erich Mendelsohn designed the Steinberg, Herrmann & Co. Hat Factory, in Luckenwalde, Germany, “bridging expressionism with the ‘forthrightness of American industrial architecture.’”^26^ Attracted by its emerging industrial infrastructure, Mendelsohn traveled to the United States in the 1920s and took a series of photographs of industrial architecture that were depicted in his 1926 photographic essay *Amerika: Bilderbuch eines Architekten*.^27^ Among the 77 powerful images of new industrial architecture in the section entitled “Das Gigantische” (“The Gigantic”), are several photographs of grain elevators, captured at dramatic angles that emphasized the plasticity of their forms and the enormity of their scale (Figs. 3, 4 and 5).

**Fig. 6:**
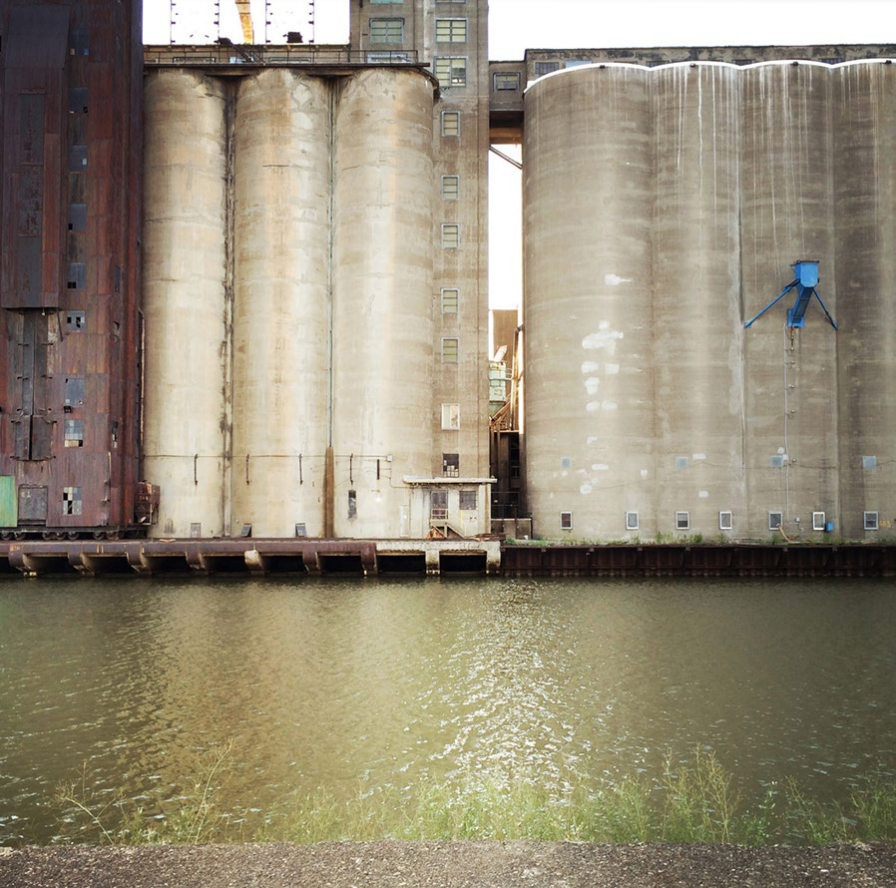
Grain elevators. Silo City. Buffalo, New York (Photo by author)

**Fig. 7:**
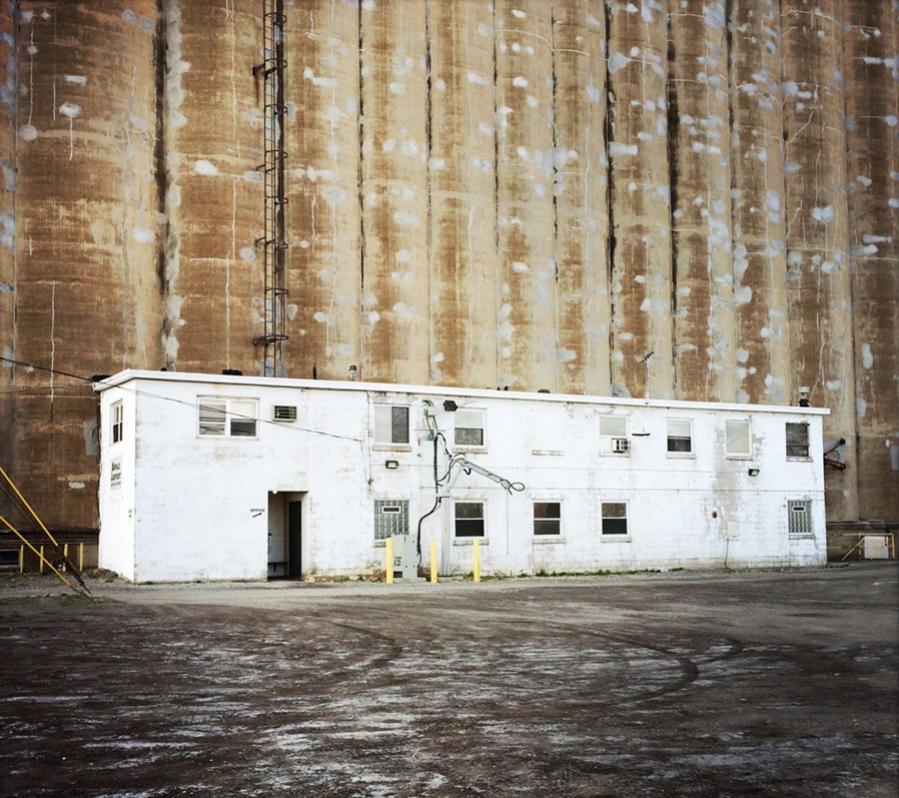
Grain elevators. Silo City. Buffalo, New York (Photo by author)

While Gropius published images of daylight factories in the *Jahrbuch des Deutschen Werkbundes* series, Mendelsohn only featured images of several elevators from his trips to New York, Chicago, Detroit, and Buffalo.^28^ Swiss-French architect Le Corbusier wrote in *Vers une Architecture* (*Toward an Architecture*) in 1923, “We have the American grain elevator and factories, the magnificent first fruits of the new age. The American engineers overwhelm with calculations our expiring architecture.”^29^ As Steiner writes, “Le Corbusier’s conclusion was that the inferior work of American architects should be passed over in favor of the honest labors of engineers in order to extract the principles of a new architecture.”^30^ To support his claim, Le Corbusier featured a photograph of Buffalo’s exposed-steel-bin Dakota Elevator, a photograph that he might have borrowed from Gropius.^31^ That same year, German-American architect Walter Curt Behrendt argued about the explicit influence of American industrial architecture on modern German architects in *Der Sieg des Neuen Baustils*:

[…] it was the example of America that gave the impulse to the German architects when they first tried to clarify the problem of structure. […] [T]his impulse did not originate in the skyscraper […] but the simple structures of industrial buildings such as grain elevators and big silos […] These examples of modern engineering, designed for practical use only, and obviously without any decorative assistance from an architect, made a deep impression by their simple structure reduced to basic forms of geometry such as cubes and cylinders. They were conceived as patterns exemplifying once more the essence of the pure form of use, gaining its impressive effect from its bare structure.^32^ (Figs. 6, 7).

The powerful character of the grain elevators and daylight factories were seen under a modern lens by English academic Reyner Banham, who had trained with one of the eminent historians of classic modernism, Nikolaus Pevsner, and taught at the School of Architecture and Planning at the University at Buffalo between 1976 and 1980, after leaving his position at University College London for his new tenure in Buffalo. During his time at the University at Buffalo, Banham worked on a scholarly project on American industrial architecture that developed into a combination of historical research, archeology, hands-on engagement, seminar instruction, and intense photographic studies, resulting in his book *A Concrete Atlantis* of 1986.^33^ Definitely, Banham understood Buffalo’s grain elevators as part of the city’s “original splendour,” as well as a crucial polemical tool in the articulation of early twentieth century modern architecture. While “the impact of Buffalo on Banham’s work remains relatively unexamined, […] Banham claimed that the ‘quintessence of European Modernism was rooted in the ‘dialectical confrontation between sculptural forms [of the grain elevators] and gridded space [of American factories].’”^34^

**Fig. 8:**
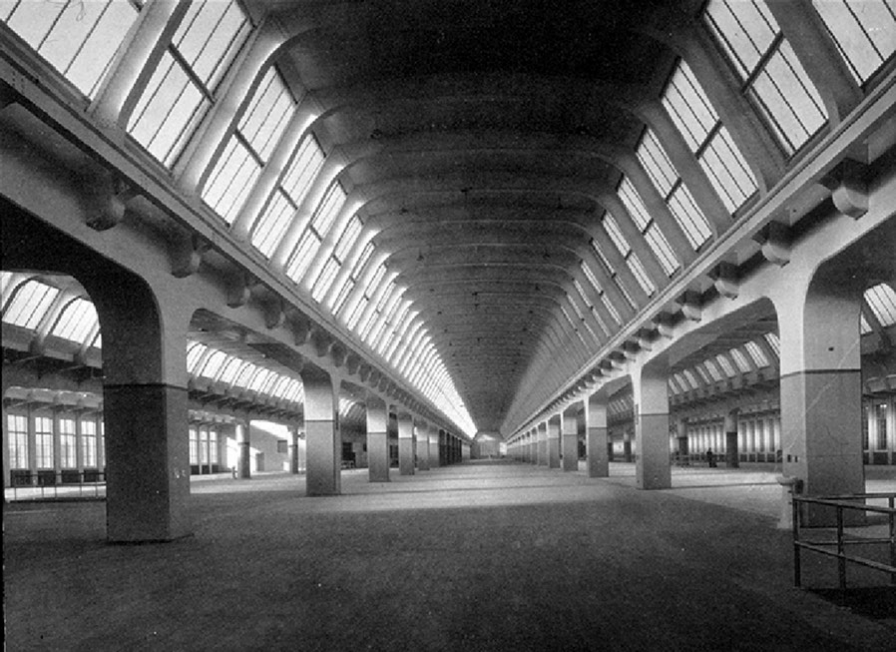
Ford Engineering Lab. Dearborn, Michigan. Architect: Albert Kahn (Photo by unknown author)

As anticipatory artifacts, the silos and daylight factories in Buffalo had influence beyond their temporal or cultural foundations, and impacted the development of an architectural grammar of exposed material, clear, and forceful geometry, and functional clarity. The ‘gridded spaces’ of the daylight factory typology provided a vocabulary of broad, open and variable floor plans and extensive glazing for natural light and ventilation, while the forceful massings of the grain elevators inspired more expressive forms of early modernism, ultimately defining Le Corbusier’s work. Through the influence of early twentieth century European architects and theorists, the grammar of the grain silos and the vocabulary of the daylight factories evolved into the language of modern architecture. Banham’s work recognized the iconic impact and persistent influence of these structures more than a century after their pragmatic ascendancy in the nineteenth century (Fig. 8).

## Material memory and place identity: reconsidering Buffalo's post-industrial ecosystems

In rethinking the decisive role of Buffalo’s post-industrial structures, a differentiation between the relevant and the prescindible may reveal the successive layers of a city’s built history, and ultimately, the value that historical constructions provide to the place identity as material narratives. The hierarchical interaction between the old and the new can take place through myriad tactics. Successful strategies for harnessing the tension between old and new may result in a powerful coexistence between the successive layers of a city’s history. The physical qualities of a city’s architecture constitute its material portraiture, which is experienced physically through multisensory engagement. The experience that emerges through these qualities contributes to an unequivocal character rooted in the city’s archaeology. As a palimpsest of the city’s material memory, post-industrial structures represent a critical chapter of the city’s core identity. The incorporation of a city’s material legacy into the design brief of new interventions and developments supports its material continuum.

**Fig. 9:**
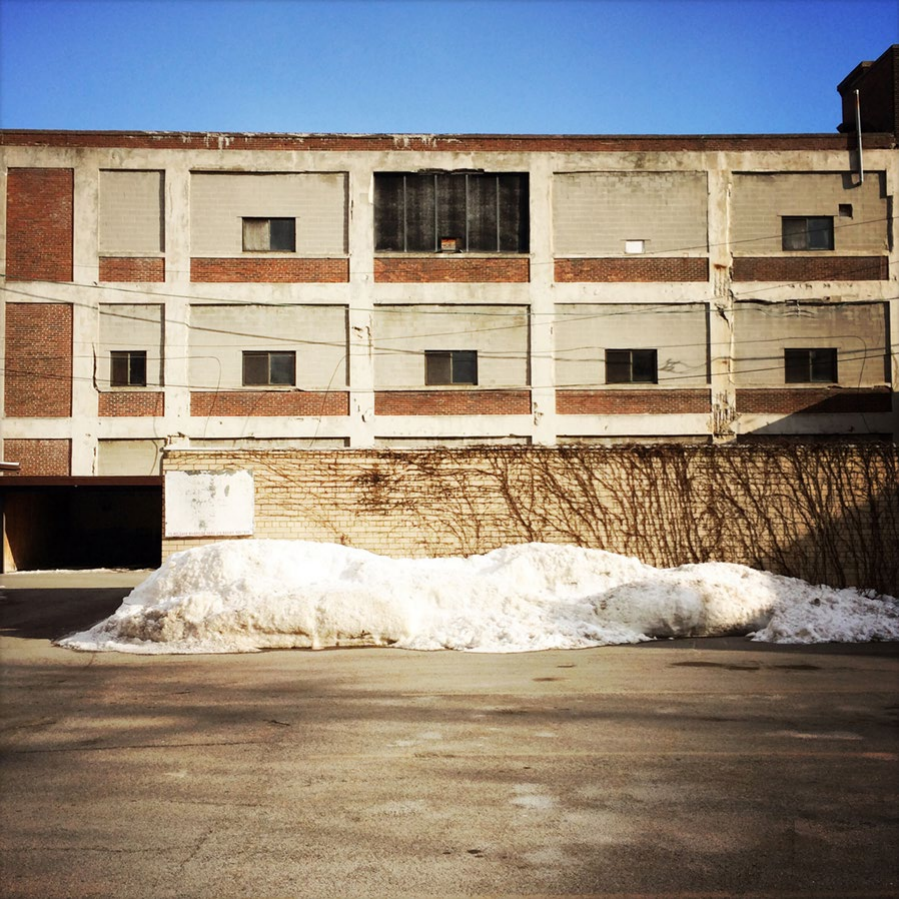
Industrial building. Buffalo, New York (Photo by author)

**Fig. 10:**
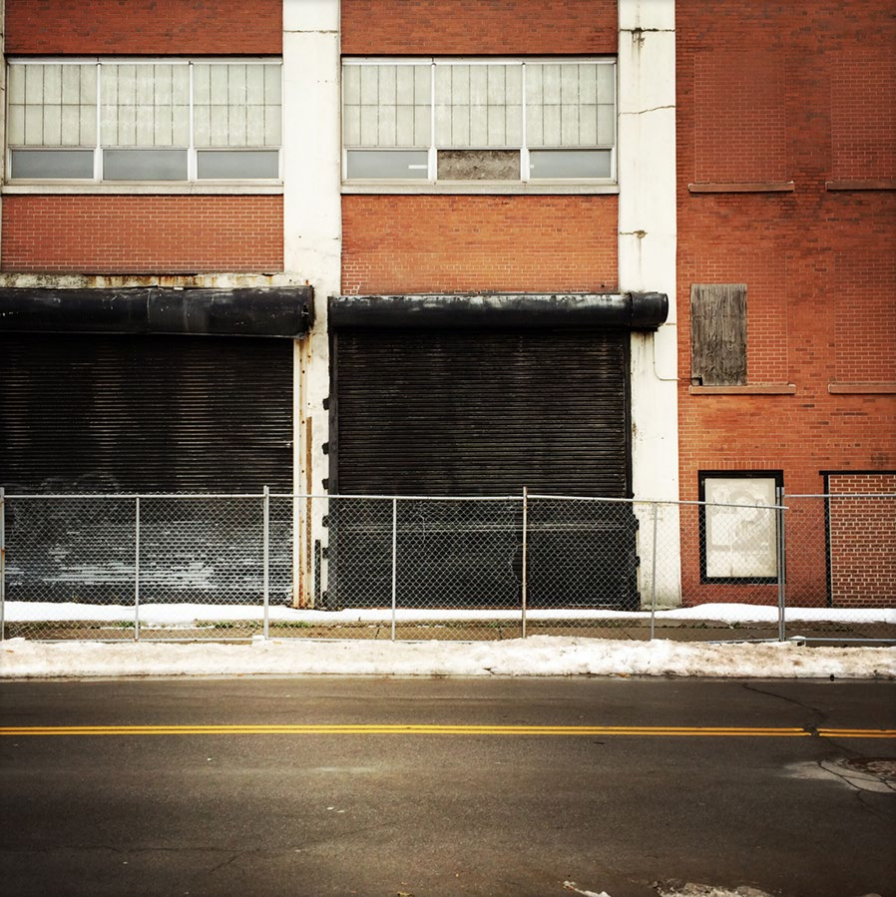
Trico Plant No 1. Buffalo, New York (Photo by author)

In disregarding its material memory, the city loses its foundations. Uprooted constructions lead the way to banal forms of identity. This consideration is especially delicate in Rust Belt cities, where industrial structures embody a distinct material legacy, the preservation of which is not always certain, due partially to their purely functional origin. This origin occasionally resulted in geometries that pose specific challenges for reuse, as buildings designed around the parameters of grain storage may be hard-pressed to accommodate new functions. In some instances, the unique forms of such structures, and their distinctive visual identity can lead to ‘ruin-porn.’ Dismissing the iconic condition of post-industrial structures creates a precarious horizon in terms of history, culture, identity and, ultimately, economic and social progress. Misunderstanding the historic and architectural prominence of these vacant structures leads to their irreversible loss. The combination of the loss of local historical and cultural identity, and the implementation of generic solutions results in a condition of urban anonymity and cultural homogeneity that debilitates any effort to reconstitute or develop a distinctive sense of identity.^35^ (Figs. 9, 10).

In addition to the dimensions that time and matter incorporate into the identity of a city’s architectural narrative, the reuse of architectural resources impacts the ties between architectural heritage and progress. Under sedulous preservation and reintegration plans, brick, concrete, and steel assemblies can yield fertile ecosystems that redraw the present and future of these structures, and their capacity to stimulate new forms of occupation.^36^ The need for investment and growth in post-industrial cities around the Great Lakes region can meet a committed consideration in reuse policies of post-industrial structures. Beyond tax considerations, the fertile environment that these vacant structures offer could frame new forms of sustainable growth while preserving and consolidating a strong material identity and, paradoxically, their immaterial memory.

Counterposing an alternative to program obsolescence, Luis Pancorbo and Inés Martín-Robles write that “ruins make visible the tectonic quality of those architectural ‘organisms’ and they therefore bring us closer to understanding their constructive process. […] The ruin of those buildings may be produced through the principle of dismantlement, not via the principle of erosion.”^37^ Pancorbo and Martín-Robles suggest how new ecosystems can emerge from the supporting frame configured by post-industrial sites. The authors state, “While the resilience of the traditional city is confined in purely cultural terms, the survival of the American industrial city is headed towards its mutation into a new, post-urban model which we could tentatively define as a ‘new technical landscape.’ This new environment, necessarily a hybrid of that which is human and that which is not human, gains similar characteristics to Gilbert Simondon’s ‘associated milieu’; it becomes an element which negotiates between technical elements and living systems.”^38^ Pancorbo and Martín-Robles reflect on how these ruined industrial landscapes might “become the support for new ecosystems which make us think about post-urban fertility,” despite current considerations that make us think of such environments as waste. The authors add: “It is the frame on which to build a hybrid, anthropogenic environment which rebalances the relationship between technical, cultural, and biological elements.” Pancorbo and Martín-Robles conclude with a specific proposal for Rust Belt cities in the form of questions: “Could the Rust Belt cities become a laboratory for the development of this new type of urban design and of new, adaptive architectural practices? Are we at the threshold of new types of colonization of this post-urban American environment?^39^

Certainly, these environments could turn into hybrid solutions that balance heritage revitalization and new construction merging technical, cultural, and biological mediums. The resulting conditions would require an explicit understanding of architectural production based on the reuse of post-industrial structures adapted to integrate contemporary practices in the context of post-industrial material, historic, infrastructural, and economic legacies. A combined “hybrid anthropogenic environment” would provide a fertile medium in which architectural production could prove to be more sustainable while preserving material elements which embody identity factors. These hybrid environments would support economic conversion and growth as active agents within their urban fabrics. Post-industrial infrastructures would thus consolidate their material continuity with their local historic-cultural legacy, performing as a distinct cultural, ecological, and economic motor for their future.

The reconstruction of Rust Belt cities necessarily involves the re-use of existing industrial structures as part of sustainable strategies that, in turn, contribute to the preservation of valuable physical attributes linked to the material character of the city. Pancorbo and Martín-Robles write that "the connection between the concepts of ruin and construction as a dialectic pair encompasses a finite linear process. […] To move away from any temptation to aesthetically romanticize ruins, it would be more beneficial to think of this process as cyclical.”^40^ The preservation of existing structures, and subsequently of its material memory, is only possible through some degree of imbrication between the vacant industrial structures and future reuse strategies that focuses significantly on material qualities and the memory they embody. Reconstruction thus cannot be considered a material *tabula rasa*, but from the organization of foundational strategies for material continuity. The connection between past and present can only be reinforced through the consideration of material memory in our cities that address continuities between foundational actions and future interventions. As stated by Pancorbo and Martín-Robles, “if construction colonizes ruins, the latter would not continue its path towards a slow fade into inexistence but would follow the path dictated by the new work that has colonized it. This fact supposes the survival not only of immaterial memory but also of material memory, within the new cycle of ruin which the new construction will be required to face once it is finished.”

As a large repository of vacant post-industrial structures, the city of Buffalo offers the possibility to renew its vacant industrial structures into sustainable and identifiable environments. Reformulated structures can become once again active parts of the urban fabric, their “obsolescence […] always unfinished and [their] historic value […] forever variable.” Of the two typologies discussed, the material constitution of daylight factories, a combination of brick, concrete, steel, and timber, combined with a regional commercial and residential vernacular of low-rise brick and timber structures, constitute the dominant materiality of the old city. While the great cylindrical forms of the grain elevators haunt the shores of Buffalo’s harbor and remain strong icons for debate, the low, long figures of the daylight factories reside throughout the city’s neighborhoods, at times bordering entire city blocks. Their ambitious scale and geographical dispersion throughout the city make the daylight factories one of the primary purveyors of the city’s material memory.

Two examples illustrate this point. The Trico Plant No 1 is a representative example of recent strategies in the functional reincorporation of industrial structures into the active identity of the city. The Trico complex consists of several daylight factories built in five stages between 1880 and 1954. It occupies almost an entire city block between Washington and Ellicott streets, on the edge of the Buffalo Niagara Medical Campus. The Trico business was once the largest employer in Buffalo and it continued to operate in the building until 1998. After years of neglect, the large complex is currently being repurposed by the developer Krog Corporation, which recognized the iconic presence of its structure and the singular materiality of its brick-and-concrete gridded façade. Krog is seeking to complete the $87 million project, which includes condominiums, apartments, parking, offices, and education spaces, by 2023. The new complex is posited to become a social, economic, and architectural beacon upon its completion.

**Fig. 11:**
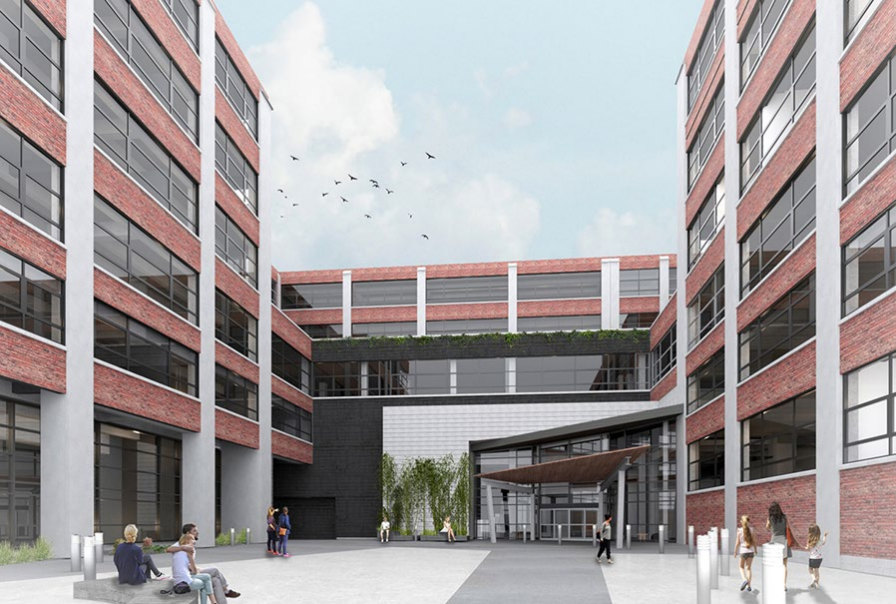
Trico Plant No 1. redevelopment. Buffalo, New York (Courtesy: The Krog Group)

The Pierce-Arrow factory complex is another example. The massive structure was designed by Albert Kahn in 1906 as a series of three-story administration and manufacturing buildings. Built mostly of reinforced concrete with brick façades with diagonal sandstone applications, the complex can be considered one of the earliest examples of daylight industrial structures along with the Packard 10 facility that Kahn built in Detroit, MI. The Pierce-Arrow complex was in full use until 1938. In the following years, the complex housed small companies that occupied the subdivided buildings. While the Pierce-Arrow complex is not subject to an integral renovation, as the Trico Plant currently is, different investments and initiatives by local developers Rocco Termini and Kanaka Partners are updating and restoring Kahn’s massive edifice to accommodate apartments and facilities attracting tenants and users to the iconic structure. These structures offer a singular material character which is an indelible part of the city’s identity. The corporations that participate of their refurbishing recognize not only the cultural and historical, but also the economic relevance of preserving the material memory held by these buildings.^41^ Revisiting these cases offers an opportunity to illustrate how “architecture faces a historical-cultural evolution which very often takes the form of a spiral with its periodic comings and goings and the continuous revisions of previous experiences."^42^ (Fig. 11).

The successful continuity of industrial structures in Buffalo shouldn’t be about aestheticizing industrial ruins through superficial forms of ‘ruin porn,’ as these are “sterile in terms of architectural design”^43^ but about implementing reuse strategies that promote physical integration into the on-going cycle of construction that contributes to define the material identity of the city. Hybrid strategies that combine industrial structures with new interventions facilitate the creation of material ecosystems that incorporate future aspirations in the continuum of the city’s material memory.

## Credible identity: Buffalo’s post industrial material ecosystems as soft power

The identity constructed with a city’s material memory helps shape the economic performance of these places. Attracting new investments that seek to avoid generic enclaves adds to—and benefits from—cities with a strong identity. As explored by Gail Dexter Lord and Ngaire Blankenberg, such identity relates directly to the notion of soft power.^44^ The authors discuss how soft power defines the ability to shape the preferences of others through appeal and attraction, that is, to attract and co-opt, rather than coerce—which would be considered hard power. The specific identity conferred by their material experience makes post-industrial cities different and recognizable, infused with the very qualities that speak to the strength of the city’s potential soft power beyond their municipal limits. If a recognizable identity defines how a city plays its roles in the competitive scenario of contemporary cities, then a city’s identity can impact the dynamics of potential investment and subsequent growth.^45^ Understanding the post-industrial material legacy is key to rethinking, redefining, reusing, and projecting, existing structures into a future that specifically addresses place-making as a revitalizing strategy with an ultimate presence in the larger scope of American cities. Architectural identity constitutes an instrumental component of such power, and helps define a general perception—positive or negative—of the contextual relevance of the city. Such non-coercive currency is defined through political values, foreign policies, as well as culture and heritage.

One condition is common to all currencies in discussing soft power: time is a necessary factor in generating credibility. The credibility of time is embodied in the material memory conveyed by the character of the urban fabric. Recognizing the relevance of such memory—and the valuable quality of historic validation associated with it—leads to a position such that the material memory of a place becomes a strategic legacy, offering a seminal agency for soft power. The partial or total destruction of the existing built environment implies the loss of the place’s history, and ultimately, the disappearance of what only time can build: its character and genuine identity as a recognizable factor. The character of a place resides in many different architectural values, but materiality is a critical part of the experienced connection between that time, identity, and credibility.^46^

The potential protection, reuse, and integration of vacant industrial buildings emerges as a seminal part of any architectural and urban agenda in Rust Belt cities, not only for an adequate preservation of their historic infrastructure, but also for the consolidation of their material identity. As the reinforcement of the character of the city’s built heritage ultimately contributes to the social well-being and economic prosperity of the area, place identity and intrinsic soft power gain strategic relevance.

The identity of a city is directly related to its material experience. The materiality of structures speaks to the qualities of their spaces and their lasting presence in our memory. Materiality becomes an opportunity to integrate past heritage with new architectural interventions, implying the possibility of cyclical renovation in a context of reinforced identity. Post-industrial sites offer fertile cases where preservation and intervention may go hand in hand, producing active contexts for architectural development as seen in the cases of the Trico Plant No.1 or Pierce-Arrow complexes. Post-industrial structures offer material conditions that help redefine the urban and architectural character of a place. The role of such heritage constitutes a negotiation between ruin and construction, a process of reiterative cycles that reconfigures the material landscape to make change possible.

In the current context, where post-industrial infrastructures are to be reconsidered, a new kind of ecology can emerge with subsequent investment and growth responding appropriately to the favorable medium.^47^ “If ruin happens at a greater scale, as occurs in the case of Detroit and other locations in the North American Rust Belt, the palimpsest between ruins and new works could span the entire fabric of the city, forming a new urban landscape.”^48^ As vacant structures merge with new interventions in a compounded palimpsest, the architectural and urban landscape improves its reusable potential while taking advantage of an unmistakable material identity.

Buffalo offers opportunities for the prosperous reintegration of its industrial legacy into new architectural ecosystems, awaiting the moment to become fertile again. The reincorporation of industrial structures into the flowing cycle of functioning architecture provides a milieu for new urban ecologies, renovated investment, and urban life, with the capacity to consolidate the city’s unequivocal identity and ultimately its leading role in cultural, economic, and social terms. The grain silos, Trico Plant No 1, and Pierce-Arrow buildings exemplify hybrid systems that reemerge in the context of a new economy, and illustrate how their material ecosystems can be reactivated instead of demolished. Reintegrated into the urban ecology, these projects can contribute to the consolidation of material memory within the future forms of Buffalo’s identity.

The hybrid ecosystems that result from these interventions in Buffalo’s industrial structures offer strategic opportunities for social and economic growth. Vacant industrial spaces are a rare opportunity for new architectural ecosystems to thrive in continuity with the city’s past. The material operations developed within Buffalo’s industrial architecture, strengthens the city’s character, and ultimately favors positive migration and investment, creativity, and growth. Among architectural resources, the forgotten structures scattered throughout Buffalo provide identity as material environments with the capacity to activate a shift from a past marred by loss to a future founded in reconciliation, promoting civic pride, authenticity, and communal identity. Buffalo’s material legacy is an irreplaceable condition of the city’s historic legacy. Enhancing the bonds between its historic and contemporary interventions reinforces a recognizable architectural identity. Far from considering these structures symbols of failure, they should be celebrated as the bones for Buffalo’s renewed identity.

## Footnotes

1 Reyner Banham, *A concrete atlantis* (Cambridge, MA: MIT Press, 1986), 175–7.

2 Buffalo went from 580,000 in 1950, when it was the 15th largest city in the country, to 256,000 as of today, equalling its 1890’s population. See: http://worldpopulationreview.com/us-cities/buffalo-population/ (Accessed November 2021).

3 Andrew Saint, review of Reyner Banham's *Concrete Atlantis*, *Architectural Association Files* No 14 (1987): 107.

4 See: Richard Florida, "Returning to the Rust Belt," Citylab.com; August 31, 2017. https://www.bloomberg.com/news/articles/2017-08-31/why-rust-belt-natives-are-coming-home (Accessed November 2021).

5 Detroit Mayor Mike Duggan’s 2018 State of the City address is a good description of the revitalization and new initiatives in the city of Detroit, only 5 years after filing for bankruptcy. https://detroitmi.gov/government/mayors-office/state-city-2019/state-city-2018 (Accessed November 2021).

6 In November of 2017, Matthew Goldstein described in *The New York Times* the difficulties in housing reoccupation and the very slow process of recovery in the specific case of the city of Detroit. Matthew Goldstein, “Detroit: From Motor City to Housing Incubator,” *The New York Times*. November 4, 2017.

7 COVID-19-related circumstances have changed living/working locations for about one out of five Americans. See: New York Center for Migration Studies https://cmsny.org/cms-initiatives/migration-covid/ and https://www.politico.com/news/magazine/2020/05/13/how-coronavirus-could-upend-human-migration-251715 (Accessed November 2021).

8 Goldstein mentions the efforts made by three large banks, Bank of America, JPMorganChase, and especially, Quicken, a local bank started by Dan Gilbert, which has invested $2.5 billion. Matthew Goldstein, op. cit.

9 The city’s population grew to 278,349 people from 261,275 in 2010, its first such gain since the 1950 census, when the population was 580,132.

10 Florida writes: “The second [key element of place character] is economic opportunity, which is itself embedded in place character, too. Returnees emphasized the specific kind of economic opportunities that emerged in a struggling, deindustrialized city. They were drawn back by the prospect of being part of its revitalization. ‘One thing about being back here is that you are a big fish in a small pond,’ one returnee said. ‘In New York and Chicago there is no way. You are a cog in a great big machine.’” Richard Florida, op. cit.

11 One driver for growth in Buffalo has been the resurgence in the distillation and fermentation industries, with numerous craft breweries and distilleries opening, along with related merchandise stores and restaurants. In Buffalo, whose history is tied deeply to grain, this represents a form of historical reversion. At the turn of the last century, Buffalo was a major transportation hub for the movement of grain from the Midwest to the East Coast. Capitalizing on the prevalence of grain, Buffalo became home to a large number of distilleries and breweries. Unfortunately, this industry was eradicated during prohibition, and it has taken almost a century to return.

12 Not only small businesses, but also large investments such as Douglas Jemal, of Douglas Development, based in Washington D.C. This particular investor has purchased important facilities in Buffalo, such as Seneca One, Sheraton hotel, Statler City, and is making proposals to take over Central Station and the Hotel Henry, formerly known as the Richardson complex. Jemal states: “The people of Buffalo love their city and take pride in it, and that is contagious.” Jonathan D. Epstein, “Douglas Jemal Says Region Needs to Step up to Lure Tech Firms Here,” The Buffalo News, Jan 28, 2021. https://buffalonews.com/exclusive/prospectus/douglas-jemal-says-region-needs-to-step-up-to-lure-tech-firms-here/article_6ff83af8-4af5-11eb-9c1d-5fba2ad343ed.html (Accessed February 10, 2022).

13 The Keep Growing Detroit’s Garden Resource Program (http://detroitagriculture.net/our-work/garden-resource-program/) and the Buffalo’s Public Art Program, promoted by the Albright-Knox Art Gallery (https://www.albrightknox.org/community/public-art/about-public-art-initiative), exemplify this point. AKAG opened a temporary gallery space on the East Side, following a strategy initiated by the MoMA when it closed for expansion.

14 The notion of “place character” carries economic implications for authors Harrison and Florida. The idea is partially configured through the material identity of the place itself. See: Jill Ann Harrison, “Rust Belt Boomerang: The pull of place in moving back to a legacy city,” *City and Community*. Vol. 16 No 3 (September 2017): 263–283.

15 “There is no perception which is not full of memories.” Henri Bergson, *Matter and Memory* (1896) (New York: Zone Books, 1988), 33.

16 Florida defines identity as the “deep, authentic character of a place itself.”.

17 Rener Banham, op. cit., 3.

18 Quoted in: Lynda Schneekloth, *Reconsidering Concrete Atlantis: Buffalo Grain Elevators* (Buffalo, NY: The Urban Design Project/School of Architecture and Planning, 2007), 14. “The grain elevators were born to support an emerging economy of transshipment that put Buffalo and the Niagara region at the center of a national and international economy. With the loss of that position and shifts in the global economy that led to the process of deindustrialization of the northeast U.S., including Niagara, the economy has sagged and the elevators, as a symbol of the economic strength of the region, now stand as negative icons to a glorified past.” Schneekloth’s book is described by its editor as “a record of the community effort on behalf of the Buffalo grain elevators through a project by the Landmark Society of the Niagara Frontier and the Urban Design Project of the University at Buffalo/SUNY. It describes the efforts of academics, preservationists, community people and funding agencies.” Dr. Schneekloth has been one of the most active voices in the advocacy and preservation of these unique structures. Her edited book *Reconsidering Concrete Atlantis* contributed to bringing renovated attention to the grain silos in 2007.

19 Claire Schneider, *Buffalo grain elevators* (exhibition catalogue) (Buffalo: Albright-Knox Art Gallery, 1999).

20 “The new abundance made the city overflow with new materials, buildings, objects; […] nothing, no one had any connection with the former Clarice. And the more the new city settled triumphantly into the place and the name of the first Clarice, the more it realized it was moving away from it, destroying it no less rapidly than the rats and the mold. Despite its pride in its new wealth, the city, at heart, felt itself incongruous, alien, a usurper. […] And then the shards of the original splendour that had been saved, by adapting them to more obscure needs, were again shifted. […] because people wanted to reconstruct through them a city of which no one knew anything now.” Italo Calvino, “Clarice,” *The invisible cities* (1972) (New York: Harcourt Brace & Co., 1974), 107. Translated by William Weaver.

21 This typology was first conceived in America by merchant Joseph Dart and mechanical engineer Robert Dunbar between 1842 and 1843.

22 See: Keith Eggener, “A fortuitous shadow,” *Places Journal* (September 2017). The article explores what makes a work of architecture American, dealing with debates about identity in architecture. https://placesjournal.org/article/a-fortuitous-shadow/ (Accessed November 2021).

23 Hadas Steiner, op. cit., 257.

24 Walter Gropius, “Die Entwicklung moderner Industriebaukunst,” *Die Kunst in Industrie und Handel*, from the series “Jahrbuch des Deutschen Werkbundes.” (Jena: Eugen Diederichs, 1913). It is to be noted that before Mendelsohn in Germany, American architect Albert Kahn had developed intense work on daylight factories in different cities of the Rust Belt, achieving what Francis Kowsky called the ‘rich dialogue.’ Francis R. Kowsky, “Monuments of a Vanished Prosperity,” *Reconsidering Concrete Atlantis: Buffalo Grain Elevators* (Lynda Schneekloth, ed.) (Buffalo: The Urban Design Project/School of Architecture and Planning, 2007), 38.

25 See: Francis R. Kowsky, op. cit., 38.

26 “Mendelsohn showed the ability to anticipate the nascent themes of modernism in the construction of the Steinberg, Herrmann & Co. Hat Factory. […] In a writing of 1930, Paul Schmidt compared Mendelssohn’s factory to a ‘tiger swiftly seizing the functionalist position that had been ignored since the Fagus and Werkbund factories had been seized by Gropius and Meyer.’ Francis R. Kowsky, op. cit.

27 Erich Mendelsohn, *Amerika. Bilderbuch eines Architekten* (Berlin: Rudolf Mosse/Buchverlag, 1926).

28 Claire Schneider writes: “[…] Visiting Buffalo in 1924, awestruck Erich Mendelsohn wrote to his wife of their ‘childlike forms.’ He described them as ‘awkward, full of elemental power, submissive to pure need. Primitive in their function of sucking in and spitting out again […] the preliminary phase of a future world just beginning to order itself’.” Claire Schneider, op. cit. The representation of these structures through photography is a subject to be explored in itself.

29 Quoted in: Reyner Banham, op. cit., 224. Reyner Banham asked American photographer Patricia Layman Bazelon to “shoot and reshoot many of the images that would appear in *A Concrete Atlantis*. Her photographs of the grain silos and daylight factories of Buffalo constitute an archive in their own right.” Hadas A. Steiner, “Cropping the view: Reyner Banham and the image of Buffalo,” *Buffalo at the Crossroads. The Past, Present, and Future of American Urbanism* (Peter H. Christensen, ed.) (Ithaca, NY: Cornell University Press, 2020), 262.

30 Hadas A. Steiner, op. cit., 257.

31 Professor Hadas Steiner writes: “[In 1919] Le Corbusier asked Gropius if he could use some of the elevator photographs in the French architectural magazine, *L'Esprit Nouveau*. The influence might have ended here. However, in 1927, some of these images reappeared in Le Corbusier’s *Towards a New Architecture*, which rapidly achieved the reputation of the book of the new era.” Hadas Steiner, “Silo Dreams,” (Lynda Schneekloth, ed.), op. cit.

32 Walter Curt Behrendt as quoted by Reyner Banham. Reyner Banham: *A Concrete Atlantis. US Industrial Building and European Modern Architecture 1900–1925* (Cambridge, MA: MIT Press. 1986), 230–1.

33 ‘“Let us listen to the counsels of American engineers. But let us beware of American architects!’ declared Le Corbusier, who like other European architects of his time, believed that he saw in the work of American industrial builders a model of the way architecture should develop. It was a vision of an ideal world, a “concrete Atlantis” made up of daylight factories and grain elevators. In a book that suggests how good Modernism was before it went wrong, Reyner Banham details the European discovery of this concrete Atlantis and examines a number of striking architectural instances where aspects of the International Style are anticipated by US industrial buildings.” See: https://mitpress.mit.edu/books/concrete-atlantis (Accessed November 2021).

34 Hadas A. Steiner, “Cropping the View: Reyner Banham and the Image of Buffalo,” op. cit, 263. Steiner quotes Reyner Banham:, op. cit., 3.

35 A recent approval to convert one of the silos into housing in Silo City poses a risky approach to a material legacy. New investments may continue to displace the raw integrity of the existing silos, contributing to their steady loss. https://www.buffalorising.com/2019/11/big-reveal-first-residential-project-at-silo-city/ (Accessed November 2021).

36 See: Keith Eggener, op. cit. note 15.

37 Luis Pancorbo and Inés Martín-Robles, “Through the Looking Glass. Construction and Ruin,” *Boletín Académico Revista de Investigación y Arquitectura Contemporánea ETSA de La Coruña*. (La Coruña, Spain: BAC. 2017).

38 Luis Pancorbo and Inés Martín-Robles, op. cit. In this context, “milieu” is used to denote the physical environment that serves as the primary agency for change. In biology, organisms are adapted through interaction with their milieu (or material substrate); architecturally, adaptation could relate to civic, economic, and social transformations. For more on milieu and medium, see: Georges Canguilhem, “The Living and Its Milieu,” *Grey Room 39* (Cambridge, MA: MIT Press. 2001): 7–31. Trans. John Savage.

39 Pancorbo and Martín-Robles suggest a special attention to plans, such as that of Camilo Vergara’s, which proposes a landscape of ruins for Detroit, like an ‘American Acropolis’ which is progressively colonized by forest vegetation, forming a natural-artificial hybrid landscape. See: Camilo José Vergara, *American Ruins* (New York: Monacelli Press. 1999).

40 Luis Pancorbo and Inés Martín-Robles, op. cit. Following quotes are from the same source.

41 While some daylight factories were left unadorned, such as the M. Wile & Co. Factory in Buffalo (Esenwein and Johnson, 1924), most were reinforced concrete construction with decorative brick and stone facades. A good portion of the brick material milieu of the city is decorative, applied to concrete buildings. Buildings such as Trico, Pierce Arrow, Alling & Cory, Bethune Hall and Mentholatum all use some form of brick facade treatment or decorative infill. All of the buildings mentioned above have been successfully repurposed and reintegrated in the fabric of the city, and many of them appear in Banham’s work.

42 Luis Pancorbo and Inés Martín-Robles, op. cit.

43 Pancorbo and Martín-Robles state that the romantic aestheticization of ruins has led to the creation of the label 'Ruin Porn,' a digression that can be considered interesting but sterile in terms of architectural design processes.

44 See: Gail Dexter Lord and Ngaire Blankenberg, *Cities, Museums and Soft Power* (Washington, D.C.: AAM Press, 2015). Preface by Richard Florida. See also: E. Cleave et al., “The Role of Place Branding in Local and Regional Economic Development: Bridging the Gap Between Policy and Practicality,” *Regional Studies, Regional Science* 3 (1) (2016): 207–228. (R. Freestone and E. Liu, eds.), *Revisiting Place and Placelessness* (London: Routledge, 2016), 1–19.

45 Architecture can be a serious instrument in the creation of soft power in our cities. The relevance of industrial iconic architecture increases dramatically the ‘marketability’ of a city. See: Gail Dexter Lord and Ngaire Blankenberg, *Cities, Museums and Soft Power* (Washington, D.C.: AAM Press, 2015). Preface by Richard Florida. The High Line in Manhattan's west side is an excellent case that illustrates not only how the recovery of an old industrial structure into the functional dynamic of a city is possible, but also in what way the same recovery can yield a contemporary icon of remarkable social and economic success.

46 This identity is the result, among others, of the physical context, which includes the material dimension of the architecture. The built-up patina can be seen as part of the material expression of strength, authenticity, credibility, and honesty, values that every potential investor would identify as attractive in terms of soft power.

47 What Florida and Harrison emphasize in their respective texts is certainly critical—the social, family, and community aspects as major driving forces for “return migration”; but this migration as an engine for urban re-birth needs to be framed in an environment of tangible physical value. Architecture, more so if genuinely local, has the capacity to create such a frame.

48 Luis Pancorbo and Inés Martín-Robles, op. cit.

